# Effect of Various Retentive Element Materials on Retention of Mandibular Implant-Retained Overdentures

**DOI:** 10.3390/molecules27123925

**Published:** 2022-06-19

**Authors:** Atitiya Chindarungruangrat, Trinuch Eiampongpaiboon, Bundhit Jirajariyavej

**Affiliations:** 1 Residency Training Program, Department of Prosthodontics, Faculty of Dentistry, Mahidol University, Bangkok 10400, Thailand; atitiyach23@gmail.com; 2 Department of Prosthodontics, Faculty of Dentistry, Mahidol University, Bangkok 10400, Thailand; trinuch.eia@mahidol.ac.th; 3 Bundhit Jirajariyavej, Department of Prosthodontics, Faculty of Dentistry, Mahidol University, Bangkok 10400, Thailand

**Keywords:** dental implantation, polyetheretherketone, overdenture, polyvinylsiloxane

## Abstract

This study aimed to examine the retentive characteristics of each retentive element material and the effects from thermocycling using the two implant-retained mandibular overdenture model. Two stud abutments and three retentive element materials; nylon, polyetheretherketone (PEEK) and polyvinylsiloxane (PVS) were used in this study. Four tested groups, with a total of 40 overdentures, were fabricated, including a Locator^®^ abutment with nylon retention insert (NY), Novaloc^®^ abutment with PEEK retention insert (PK), Locator^®^ abutment with PVS retention insert (RL), and Novaloc^®^ abutment with PVS retention insert (RN). The retentive force (N) was measured before thermocycling, and at 2500, 5000, and 10,000 cycles after thermocycling. Significant changes in the percentage of retention loss were found in the NY and PK groups (*p* < 0.05) at 6 and 12 months for the RL group (*p* < 0.05) after artificial aging. The RN group exhibited a constant retentive force (*p* > 0.05). The tendency of the percentage of retention loss significantly increased for PEEK, nylon, and PVS silicone over time. The results of the present study implied that retentive element materials tend to lose their retentive capability as a result of thermal undulation and water dispersion. Nylon and PEEK, comprising strong polar groups in polymer chains, showed a higher rate of retention loss than polyvinylsiloxane.

## 1. Introduction

The aging population is currently a global challenge, and total edentulism, which is prevalent in the elderly and is considered as a major public health problem for the world population [1]. In 2002, the McGill consensus reported that the mandibular two implant-retained overdentures were considered as the first choice of standard care for edentulous patients [2]. Implant-retained overdentures offer superior retention and stability provided by the attachment mechanism [3]. Patients with implant-retained overdentures were more satisfied with the comfort and mastication efficiency of their dentures than those without the implants [4,5]. In order to derive satisfactory retention for the patient, various attachments have been employed, including bar, ball, stud, and magnet [6,7]. The retentive force of the attachments is obtained between the patrixes and matrixes by either mechanical interlocking, frictional contact, or magnetic forces [8]. Numerous studies have shown that adequate retention is linked to higher levels of patient satisfaction [4,5,9]. Stud attachments are popular among attachment systems due to their simplicity, less technique sensitivity, reduced cost and ease of maintenance [6,10]. The “adequate or acceptable” retention required for an attachment system remains unclear. The minimal retentive force predicted for a single individual, unsplinted attachment has been suggested to be 4 N [11]. Various retentive forces ranging from 1 to 85 N have been reported for different attachment systems in mandibular implant-retained overdenture [10,11,12]. A retentive force of around 5–7 N would sufficiently stabilize an overdenture, according to Pigozzo [13]. However, for the elderly or frail patients, retentive forces from attachment systems may be too high for overdenture wearer [14].

A change in the retentive capacity of attachment systems, which are typically polymers, is expected when the overdentures are subjected to mechanical stress, temperature and chemical reactions [15,16]. Such changes usually occur within the first year of use [17,18].

Locator^®^ is a widely used attachment system for implant-supported or implant-retained overdentures that ensures correct seating and appropriate retention [19]. It offers self-alignment and dual retention, and requires a minimal interarch space. The interchangeable nylon inserts are available in different retention values. Nylon has been used due to its elasticity and biocompatibility, but it shows extensive deformation and requires considerable maintenance [20]. There are various commercial attachment systems used in dentistry. Novaloc^®^ is a newly created attachment using high fracture toughness polymer (polyetheretherketone or PEEK). The plastic retention insert possesses high chemical and mechanical resistance [21]. Recently, Retention sil^®^ has been introduced as another material for the retentive element, however, studies of this component are limited. It contains polyvinylsiloxane (PVS), which is resiliently adapted to the attachments to ensure a resilient position of dentures. This system is considered to be both constantly retentive, easy to remove and economical [20,22]. Therefore, it may be suitable for the elderly people due to its low retentive value [22]. 

To date, many newly developed attachment systems and materials are available for overdentures. It might confuse dentists in selecting the most suitable design for their patients. Although the retentive properties of various attachment designs have been explored in prior studies, very few research studies have been conducted on the influence of retentive element materials concerning attachment retention. Furthermore, the change in retention force of these materials from thermal aging in the oral cavity has not been widely discussed. Therefore, this study aimed to examine the retentive characteristics of retentive element materials and the influence of thermocycling on their retention using two implant-retained mandibular overdenture model. The null hypothesis was that there was no difference in the retentive force for each of the three retentive element materials evaluated before and after thermocycling.

## 2. Materials and Methods

A detailed list of the attachment systems used in this study is given in Table 1. Two stud abutments; Locator^®^ (Zest Anchors Inc., Escondido, CA, USA) and Novaloc^®^ (Valoc., Möhlin, Switzerland) and three retentive element materials; nylon, polyetheretherketone (PEEK), and polyvinylsiloxane (PVS) were investigated in this study. A total of 40 overdentures were fabricated, and they were allocated into 4 groups (*n* = 10) as follows; Group 1—Locator^®^ abutment/nylon retention insert (NY); Group 2—Novaloc^®^ abutment/PEEK retention insert (PK), Group 3—Locator^®^ abutment/PVS retention insert (RL), and Group 4—Novaloc^®^ abutment/PVS retention insert (RN). Sample size calculation was performed using the Freeware G*Power, Version 3.1.9.2 [23]. The retention force of each model was measured before thermocycling and at 2500, 5000, and 10,000 cycles after thermocycling.

### 2.1. Specimen Fabrication

**Figure 1:**
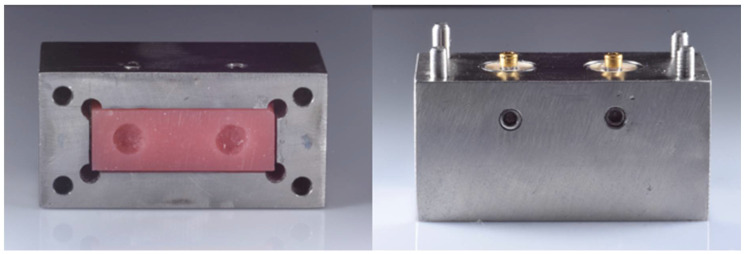
The upper and lower portions of specimens with an acrylic block.

Each specimen consisted of two parts (Figure 1). The lower portion of the specimen mimicked the patient’s mandible. A 30 mm × 60 mm × 30 mm stainless-steel block was made with two identical holes with a diameter of 16 mm, creating a distance of 23 mm apart, simulating the conventional placement of implants at an osteotomy site in the mandible [24]. It contained implant analogs (RC analog; length 12 mm; material, stainless steel; Institut Straumann AG, Basel, Switzerland) that were attached to Novaloc^®^ and Locator^®^ screw-retained abutments. The two parallel analogs were inserted and fixed in the metal block vertically using a positioning jig and dental stone (Type 4 dental stone, M dent, Bangkok, Thailand). The abutments were fitted onto the implant analogs and tightened as instructed by the manufacturers. Another 30 mm × 60 mm × 30 mm metal block was created for the upper part. To simulate the overdentures, a silicone mold was designed to be used as a template to manufacture acrylic blocks. The mixture of autopolymerizing polymethyl methacrylate (PMMA; Unifast Trad; GC Corporation, Tokyo, Japan) powder and monomers in the dough state was packed in the silicone mold under compression through the polymerization process to create the overdenture model.

**Figure 2:**
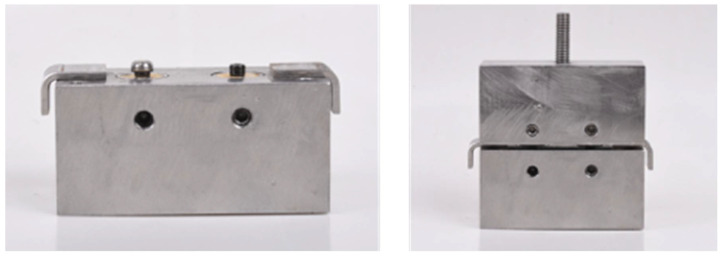
A metal spacer used to provide space between the upper and lower specimens.

For the Novaloc^®^ and Locator^®^ groups, the matrix housings and corresponding retentive element materials were embedded in acrylic blocks using direct attachment pick-up techniques. Metal housings with processing inserts were placed on the abutments. Then the acrylic block was mounted over the housings and adequate relief for the housings was verified. The metal housings were picked-up in acrylic blocks using auto-polymerized acrylic resin (PMMA; Unifast Trad; GC Corporation, Tokyo, Japan). A metal spacer (2 mm in thickness) was used to provide the space between the upper and lower blocks while the resin was polymerized, and to serve as a stop for the upper block to ensure the accurate position of every pick-up procedure (Figure 2). Finally, the processing inserts for each attachment system were removed and replaced with the appropriate PEEK and nylon inserts.

In addition, Retention sil (Retention sil 600; Bredent medical GmbH and co.KG) was used as the alternative retentive elements for the Novaloc^®^ and Locator^®^ groups (group RL and RN). Two cavities were drilled at the acrylic block to create the space of 1 mm for silicone material, and the space was applied with a primer for the silicone retention (Multisil; Bredent medical GmbH and co.KG). The retention sil silicone material was filled into the holes of in the acrylic block. The acrylic block was subsequently positioned over the abutments until the silicone is cured and the excess silicone was trimmed. The fixation of both upper and lower specimen holders could be performed reversibly on the universal testing machine (Instron 5566; Instron Ltd., High Wycombe, UK). 

### 2.2. Retention Force Measurement

**Figure 3:**
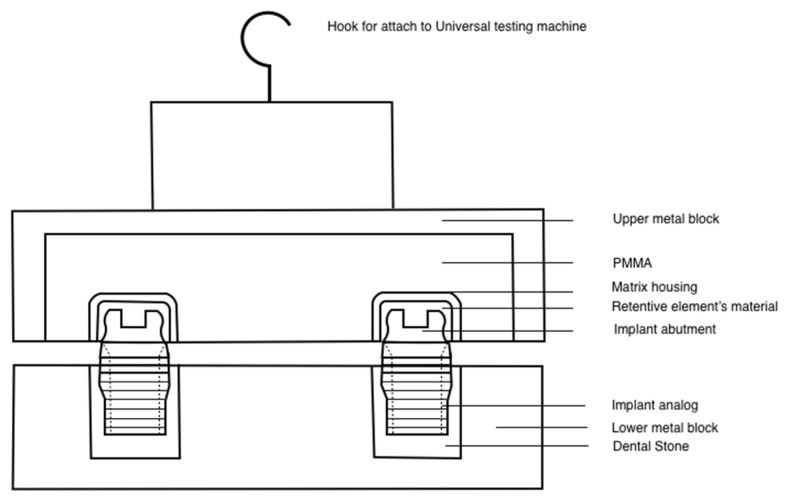
Schematics of the specimen attachment to the universal testing machine.

The retention force of the specimens was measured using the universal testing machine (Instron 5566; Instron Ltd., High Wycombe, UK). The upper block was attached to the upper moving arm of the universal material testing machine, while the lower block was secured to the machine by orienting it parallel to the horizontal plane (Figure 3). Force was applied by a centrally positioned hook connected to a 100-N load cell at a crosshead speed of 50 mm/min until complete separation of both parts under dry conditions [11,17,25]. The retention force (N) was determined by separating the acrylic block with retentive elements from the abutments. This controlled vertical force simulated the insertion and removal of overdentures and it can be compared to the prior studies in terms of prosthesis retention measurement. This value was also calculated as the percentage of retention loss over time according to the thermocycling process.

### 2.3. Artificial Aging

All acrylic blocks were artificially aged in the thermocycler (TC 400; King Mongkut’s Institute of Technology, Ladkrabang, Bangkok, Thailand) for a total of 10,000 alternating cycles between 5 and 55 °C in the water baths. The immersion time was 30 s [19]. The temperature range of 5 to 55 °C was chosen as equivalent to the temperature of foods consumed during meals while avoiding damage to oral tissues [26]. The retention force was measured using the universal testing machine under dry conditions and at 2500, 5000, and 10,000 after thermocycling where 10,000 cycles of thermocycling are correspondingly equivalent to 12 months of service in the oral cavity [27,28]. 

### 2.4. Statistical Analysis

The data were analyzed using IBM SPSS Statistics Software (IBM SPSS Statistics for Windows, Version 28.0. IBM Corp., Armonk, NY, USA). Repeated measures ANOVA were used to analyze the difference in retention values at a 5% significance level (α = 0.05). 

## 3. Results

**Figure 4:**
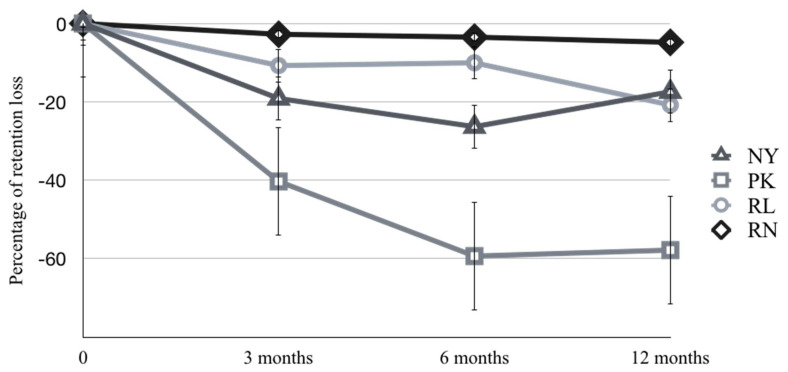
The percentage of retention loss by groups over times.

The retention forces before and after the thermocycling process are listed in Table 2. The mean values of the initial retentive force of all four tested groups were greater than those of the manufacturer’s specifications. The majority of the retentive data showed a declining trend after thermocycling. However, the retentive values of the Locator^®^ group (NY) initially decreased and regained the retention at 12-month period. The reduced retention force values of each group were calculated as the percentage of retention loss according to the differences from the initial retention values over time (thermocycling), and are listed in Table 3. Between the baseline and six months, significant changes in the percentage of retention loss were found in the NY and PK groups (*p* < 0.05) and at 12 months for the RL group (*p* < 0.05) after artificial aging from the thermocycling process. The RN group exhibited a constant retentive force (*p* > 0.05). The tendency of the percentage of retention loss was found to be higher for PEEK, nylon, and PVS silicone (Figure 4).

## 4. Discussion

Mandibular two-implant-retained overdentures that provide sufficient retention to lower the complete denture from the attachment system is considered a reliable treatment option. To achieve patient satisfaction, the minimum acceptable retention has been about 8 to 20 N [4,29]. Therefore, the initial retentive forces of tested attachment systems in this study (8.54–46.34 N) were within the range of acceptable retentive forces for the overdenture. After the simulated period of 12 months, the final retentive forces (7.83–37.28 N) were significantly lower than the initial retentive forces, but they were still sufficient to maintain an overdenture according to Pigozzo that “5–7 N would be sufficient to stabilize an overdenture” [13].

The attachment system’s materials, designs, and dimensions can impact on the retention force [10]. The retentive materials used in this study consisted of nylon or polyamide, PEEK, and PVS. Because of the varied test sets and simulated settings, the results of this study can only be compared to other studies to a limited extent. Because many factors affect overdenture retention and stability intra-orally, reproducing exact intra-oral displacement patterns is unlikely. To compare results with other studies, a linear dislodgement movement was performed on the model perpendicular to the occlusion plane at a cross-head speed of 50 mm/min to determine retentive property [11,17,25]. Retention is defined as the ability of a prosthesis to withstand dislodgment forces along the axis of insertion.

In agreement with Chung et al., they examined the retentive characteristics and longevity of implant retained overdentures, and it was suggested that the oral condition should be represented in the experiment. One of the factors to simulate oral cavity condition is thermal cycling [11]. Because the prosthodontic maintenance of implant overdentures is higher in the first year [15], 10,000 thermocycling cycles in water between 5 and 55 °C were produced to simulate the clinical use of the overdentures for one year [19,28]. 

The thermocycling process diminished the retention forces in the current study, particularly in the PK and NY groups, which lost up to 57 percent of their retention forces. However, the decrease in retention forces was more consistent in the RL and RN groups under the same condition. Nevertheless, following a period of drastically decreasing retentive force, the retentive force of the nylon retentive element of the Locator^®^ group (NY) increased at 12 months when compared with at 3- and 6-months periods. Shastry found a drop in retentive potential in Locator^®^ attachment after 5000 cycles of thermocycling [16]. Chiu investigated the influence of water temperatures on Locator^®^ attachment retention and discovered that water temperatures at 60 °C significantly reduced its retentive value [30]. Schweyen evaluated the retentive force of Locator^®^ and vinylpolysiloxane attachments after a fatigue test and thermal undulation, and discovered that the Locator^®^ showed a considerable decline in retention forces, while the PVS groups showed no change in retention forces [22]. Li investigated PEEK post-core restoration with PVS attachment systems and discovered the slightly decrease in retention force value in the strong linear relationship with cyclic time [31]. Furthermore, according to a study conducted by Arnold, the thermocycling procedure reduced the average retention force by 33% in Novaloc^®^ when compared with baseline values [32]. 

Obviously, moisture accelerates property changes in polymers. Water could be bound to polymer chains containing strong polar groups, such as the ketone group (C=O), through hydrogen bridges, and act as a softening agent [33]. The ketone group is present in both nylon and PEEK chemical structures. This chemical group effectively binds to water, then spaces the chains and reduces the mechanical characteristics of the materials. The diffusion of water throughout a polymer matrix could explain the drop in retentive force measurements observed in PEEK. Water penetrated the polymer chains, loosening the surface and deteriorating the mechanical properties [34]. 

According to the manufacturer, nylon (Dupont Zytel 101 L NC-10 Nylon; Zest Anchors Inc., Escondido, CA, USA) is an unreinforced polyamide 66 resin for injection molding. Moisture and temperature are the two most important factors affecting the characteristics and performance of unreinforced polyamide 66 [35]. Polyamides respond to moisture contents in the environment by reversibly absorbing or releasing water. Initially, water molecules diffuse into polyamide chains, forcing them apart and weakening bonds. After 12 months of artificial aging, a significant amount of water absorbed in the polymer may cause the retention insert to swell. Therefore, the gap between the abutment and the nylon insert has been minimized and could therefore be the explanation for an increase in retention force [32]. 

PVS silicone material (RN and RL groups), on the other hand, seemed to have no strong polar groups which could bind to water in its polymer chains, and it provided constant retention forces after one year of artificial aging. Despite the fact that the PVS attachment has lower initial retention values than the other three systems, among patients with dexterity problems or the elderly who may have difficulties inserting and removing the overdentures, a lower retention attachment may be preferable [22]. Another clinical advantage of the constant retention property in the PVS group is that it could be used as a cost-effective repair material. 

Nylon and PVS, which have long been used and known for their safety in biocompatibility. Katzer discovered that PEEK fiber material is biocompatible and not cytotoxic to cells after incubating it with strains of Salmonella bacterium [36]. Moreover, Peng investigated biofilm growth on the surface of PEEK and discovered that it is less conducive to biofilm formation [37].

The significant loss of retention observed at six months of both nylon and PEEK attachment in this study necessitates the replacement of the retentive insert to maintain constant and reliable retention for the overdentures. The cost of maintenance is also a factor to be considered when selecting the implant overdenture attachment systems. However, this study focused solely on the effect of temperature, and only unidirectional force was applied. Many oral factors, including masticatory loading, parafunctional habits, the number of dentures inserted and removed, the presence of saliva, and the use of a denture cleansing agent, could promote retention loss of the attachment system [12,38]. Furthermore, investigations on the effect of the materials on simulated body fluid environment and its resulting oral bacteria are crucial. Therefore, there should be further studies to simulate the other oral factors. 

## 5. Conclusions

Thermal undulation and water dispersion affects retentive element materials to deteriorate, causing them to lose their retentive potential. Nylon and PEEK, comprising strong polar groups in polymer chains, showed a higher rate of retention loss than PVS. Within this study’s limitations, the retentive value obtained from several polymeric materials, including Nylon, PEEK and PVS, decreases after one year of artificial aging. To maintain the attachment’s retentive properties, a six-month periodic recall period should be recommended. The PVS attachment system could be used to constantly repair materials for various stud abutments and for frail patients.

## Figures and Tables

**Table 1:** Materials and labeling of tested attachment systems.

Labeling	Description			
	Abutment	Retention Insert	Retentive Element Materials	Retentive Value
**NY**	Locator^®^ abutment *	Locator^®^ retention insert	Nylon or Polyamide	Blue (680 g)
**PK**	Novaloc^®^ abutment *	Novaloc^®^ retention insert	Polyetheretherketone (PEEK)	White (750 g)
**RL**	Locator^®^ abutment *	PVS retention insert	Polyvinylsiloxane (PVS)	Retention sil 600 (600 g)
**RN**	Novaloc^®^ abutment *	PVS retention insert	Polyvinylsiloxane (PVS)	Retention sil 600 (600 g)

* Novaloc^®^ and Locator^®^ regular neck abutments (RN) at 0 degrees with 2 mm gingival height were selected.

**Table 2:** Mean and SD of retentive force (N) of the tested groups at 0, 3, 6, and 12 months artificial aging.

Parameter/ Time (Months)	Retentive Force (N)			
	PK	NY	RL	RN
0	46.34 (3.72)	45.11 (4.22)	9.86 (0.97)	8.54 (0.90)
3	27.76 (4.69)	36.52 (4.53)	8.82 (1.23)	8.30 (0.83)
6	18.77 (2.19)	33.10 (3.56)	8.88 (1.09)	8.24 (0.91)
12	19.61 (3.41)	37.28 (4.42)	7.83 (1.39)	8.12 (0.80)

**Table 3:** Mean and SD of Percentage of retention loss of tested groups at 0, 3, 6, and 12 months artificial aging.

Parameter/ Time (Months)	Percentage of Retention Loss			
	PK	NY	RL	RN
0	0 ^a^	0 ^c^	0 ^e^	0 ^g^
3	40.33 (7.10) ^a^	19.15 (5.11) ^c^	10.75 (6.11) ^e^	2.76 (2.12) ^g^
6	59.36 (4.57) ^b^	26.35 (7.52) ^d^	10.04 (5.94) ^e^	3.49 (3.33) ^g^
12	57.83 (5.36) ^b^	17.44 (4.94) ^c^	20.82 (10.20) ^f^	4.82 (3.17) ^g^

*p* Values are from repeated measures ANOVA. The different lowercase letter in columns indicates significant differences (*p* < 0.05).

## Data Availability

The data presented in this study are available on request from the corresponding author.
